# Knowledge and Preference Towards Mode of Delivery among Pregnant Women in the United Arab Emirates: The Mutaba’ah Study

**DOI:** 10.3390/ijerph18010036

**Published:** 2020-12-23

**Authors:** Rami H. Al-Rifai, Iffat Elbarazi, Nasloon Ali, Tom Loney, Abderrahim Oulhaj, Luai A. Ahmed

**Affiliations:** 1 Institute of Public Health, College of Medicine and Health Sciences, United Arab Emirates University, Al Ain P.O. Box 17666, UAE; ielbarazi@uaeu.ac.ae (I.E.); nasloona@uaeu.ac.ae (N.A.); aoulhaj@uaeu.ac.ae (A.O.); luai.ahmed@uaeu.ac.ae (L.A.A.); 2 Zayed Centre for Health Sciences, United Arab Emirates University, Al Ain P.O. Box 17666, UAE; 3 College of Medicine, Mohammed Bin Rashid University of Medicine and Health Sciences, Dubai P.O. Box 505055, UAE; tom.loney@mbru.ac.ae

**Keywords:** maternal health, mode of delivery, knowledge, United Arab Emirates

## Abstract

Background: The rate of cesarean section (CS) is growing in the United Arab Emirates (UAE). Pregnant women’s knowledge on the mode of delivery, factors associated with lack of adequate knowledge, and preference towards CS delivery were investigated. Methods: Baseline cross-sectional data from 1617 pregnant women who participated in the Mutaba’ah Study between September 2018 and March 2020 were analyzed. A self-administered questionnaire inquiring about demographic and maternal characteristics, ten knowledge-based statements about mode of delivery, and one question about preference towards mode of delivery was used. Knowledge on the mode of delivery was categorized into “adequate (total score 6–10)” or “lack of adequate (total score 0–5)” knowledge. Crude and multivariable models were used to identify factors associated with “lack of adequate” knowledge on the mode of delivery and factors associated with CS preference. Results: A total of 1303 (80.6%) pregnant women (mean age 30.6 ± 5.8 years) completed the questionnaire. The majority (57.1%) were ≥30 years old, in their third trimester (54.5%), and had at least one child (76.6%). In total, 20.8% underwent CS delivery in the previous pregnancy, and 9.4% preferred CS delivery for the current pregnancy. A total of 78.4% of pregnant women lacked adequate knowledge on the mode of delivery. The level of those who lacked adequate knowledge was similar across women in different pregnancy trimesters. Young women (18–24 years) (adjusted odds ratios (aOR), 3.07, 95% confidence interval (CI), 1.07–8.86) and women who had CS delivery in the previous pregnancy (aOR, 1.90, 95% CI, 1.06–3.40) were more likely to be classified with a lack of adequate knowledge. Age (aOR, 1.08, 95% CI, 1.02–1.14), employment (aOR, 1.96, 95% CI, 1.13–3.40), or previous CS delivery (aOR, 31.10, 95% CI, 17.71–55.73) were associated with a preference towards CS delivery. Conclusion: This study showed that pregnant women may not fully appreciate the health risks associated with different modes of delivery. Therefore, antenatal care appointments should include a balanced discussion on the potential benefits and harms associated with different delivery modes.

## 1. Introduction

Previous research has shown that the World Health Organization (WHO) recommendation to limit population-based cesarean section (CS) rates to 10–15% is associated with decreases in maternal, neonatal, and infant mortality [1]. However, in the last three decades, there has been an increase in CS rates in both developed and developing countries [2]. This rise in CS rates places a burden on public health services, especially when CS is performed unnecessarily, leading to economic and service deficiencies and consequently poorer maternal and child health outcomes [3,4]. Compared to vaginal delivery, CS is documented to be associated with a higher risk of, but not limited to, postpartum infection, urinary tract infection, pain, headaches, anesthetic complications [5,6], maternal death [7], and postpartum depression [8].

Patients involved in the clinical decision-making process and provided with customized care have been shown to make better choices regarding their health [9,10,11]. Women’s knowledge and awareness about the needs and complications of CS was shown to reduce the rate of elective CS deliveries [12,13]. Educating women and raising their awareness may contribute to a reduction in unnecessary CS deliveries, which will thereby reduce maternal and newborn morbidity and mortality. Investing in this area of patient care is important to define the gaps, to raise community and patients’ awareness, and to enable them to make better informed choices.

Although the United Arab Emirates (UAE) has achieved targets of the Millennium Development Goals (MDGs) number 4 [14] on reducing the under-five mortality by two-thirds, and number 5 on reducing the Maternal Mortality Ratio (MMR) by three-quarters, between 1990 and 2015 [15], the rates of CS are reported to be above the WHO recommended rates of 10–15% [2]. In 2017, a community-based survey revealed that 30.2% of women in the Abu Dhabi Emirate, the capital of the United Arab Emirates (UAE) underwent CS [16]. There are limited studies exploring the sociodemographic and cultural factors associated with obstetric outcomes such as mode of delivery in the UAE and the region [17]. Until today, knowledge on the mode of delivery and the factors contributing to the choice of CS among women and practitioners in the UAE have not been fully investigated. Determining pregnant women’s knowledge on the mode of delivery, as well as the factors that may influence their preference for CS delivery, will guide policymakers and healthcare providers in designing effective policies and interventions in order to help meet the target of decreasing CS deliveries in the UAE for better maternal and child health outcomes. The objectives of this study were to investigate the (i) level of maternal knowledge on delivery mode; (ii) demographic and maternal characteristics associated with a lack of adequate knowledge on mode of delivery; and (iii) preferred mode of delivery and factors associated with preference towards CS delivery for the current pregnancy among pregnant women from the Emirati population.

## 2. Materials and Methods

This is a cross-sectional analysis of the baseline data from pregnant women who participated in the Mutaba’ah Study between September 2018 and March 2020. The Mutaba’ah Mother and Child Health Study is an ongoing prospective cohort study in Al Ain city, UAE. All pregnant women from the Emirati population who were 18 years and above, were residents in Al Ain, and were able to provide informed consent were eligible to participate in the study. Pregnant women were consecutively recruited at any time throughout their pregnancy during their antenatal care (ANC) visits at three major health institutions in the city. Pregnant women completed two sets of self-administered electronic questionnaires. More details about the Mutaba’ah Study are available elsewhere [18].

Data for this analysis was extracted from two baseline questionnaires. No questions were repeated in the two baseline questionnaires. The first questionnaire collected information on the women’s demographics (age, education attainment, employment status, and husband’s education attainment) and maternal characteristics (gestational trimester, parity, gravidity, mode of last delivery, history of pregnancy-related events).

To measure adequacy of maternal knowledge and awareness, the second questionnaire included ten statements inquiring about mode of delivery. These ten statements were previously validated and used elsewhere [19]. For scoring knowledge statements, “1” point was given to each correct response while “0” points were given to each incorrect and “I don’t know” answers. The overall maternal knowledge score was then categorized as “lack of adequate knowledge” (outcome of interest), defined as scoring at most 5, or “adequate” knowledge, defined as scoring at least 6, on a scale of 10. The questionnaire also asked women about their preferred mode of delivery for the current pregnancy.

Descriptive and inferential analyses were performed. In descriptive analyses, pregnant women were described according to the measured demographic and maternal characteristics, and according to the adequacy of knowledge on the mode of delivery (lack of adequate vs. adequate). The frequency and proportion of pregnant women according to their preferred mode of delivery for the current pregnancy by their measured characteristics were also quantified. Continuous variables were quantified as means and standard deviations (SD), while categorical variables were quantified as frequencies and proportions. For continuous variables, Student’s *t*-test was used to determine differences between group means. For categorical variables, Pearson Chi-square test was used to determine differences between group proportions.

In inferential analyses, univariate and multivariate logistic regression models were used to quantify the strength of association between the measured characteristics and a lack of adequate knowledge on the mode of delivery. The multivariable model included age (as a continuous variable), gestational trimester, women’s education, history of eventful pregnancy (e.g., gestational diabetes (GDM)), and mode of delivery in the previous pregnancy.

To quantify the strength of association between the measured characteristics and preference for CS delivery as the preferred mode of delivery for the current pregnancy, univariate and multivariate regression models were used. The multivariable model included age (as a continuous variable), employment, parity, adequacy of knowledge on mode of delivery, and history of CS delivery. Due to the strong collinearity with gravidity (Variance Inflation Factor >10), parity was prioritized to be included in the multivariable model due to the stronger association in the crude model.

Crude odds ratio (OR) and adjusted odds ratios (aOR) with 95% confidence intervals (CI) were reported. Statistical significance was defined by a p-value less than or equal to 0.05 and CI.

All analyses were performed by the IBM SPSS software v26. We followed the STROBE guidelines for reporting cross-sectional studies [20].

This study was approved by the United Arab Emirates University-Human Research Ethics Committee (ERH-2017-5512, 18-03), Al Ain Hospital Research Committee (AAHEC-03-17-058), and the Tawam Hospital Research Committee (IRR-494). All participants provided informed written consent prior to participation.

## 3. Results

### 3.1. Profile of the Study Population

During the study period, 1617 pregnant women were approached and 1303 (80.5%) answered the study survey. The mean age of pregnant women was 30.6 years (±5.8 SD). At the time of the survey, 57.1% of pregnant women were ≥30 years old, 53.1% attained a two-year diploma or above, 68.9% were unemployed, and 54.4% were in the third gestational trimester. Almost a quarter (23.4%) of pregnant women were nulliparous while nearly 70.0% had at least one lifetime pregnancy. Of the pregnant women, with an average of 3.0 children (±1.8 SD), 20.8% reported having CS delivery in the previous pregnancy. Furthermore, more than three-quarters (77.0%) of women who ever been pregnant reported ever experiencing at least one eventful pregnancy (e.g., ever experienced GDM or miscarriage). Moreover, 9.4% of pregnant women preferred undergoing CS delivery for their current pregnancy (Table 1).

### 3.2. Knowledge Towards Mode of Delivery

Table 2 presents the ten knowledge-based statements inquiring about the mode of delivery. Out of 10, the mean knowledge score was relatively low (3.7 ± 2.2 SD). Only one pregnant woman answered all the statements correctly. More than three-quarters (76.1%) perceived that CS is less painful than vaginal delivery, 83.2%, 81.2%, and 83.9% expected that bone fracture is impossible in CS delivery, respiratory disorders are less likely, and maternal hemorrhage is less likely to happen in CS compared to vaginal delivery, respectively. However, 62.4% of pregnant women were knowledgeable that greater maternal complications are associated with CS than vaginal delivery. More than half (59.2%) of the pregnant women were aware that CS delivery is reasonable in breech presentation (Table 2).

**Figure 1:**
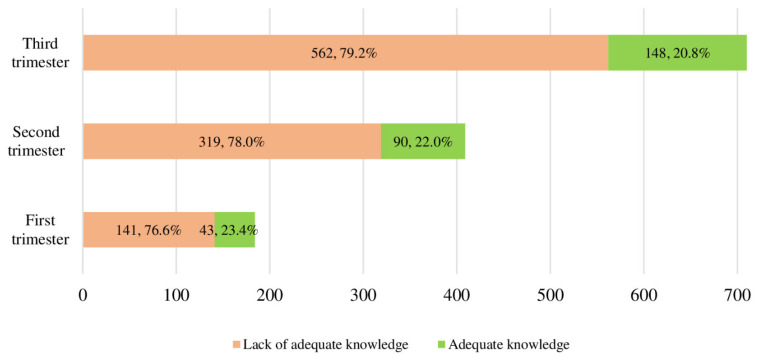
Comparison of knowledge towards mode of delivery among different women at different pregnancy trimesters (*p* = 0.734).

Categorizing pregnant women into having a lack of adequate (total score = 0–5) and adequate (total score = 6–10) knowledge, revealed that 78.4% of pregnant women lacked adequate knowledge on the mode of delivery (Table 2). Surprisingly, the proportion of pregnant women with a lack of adequate knowledge on the mode of delivery was similar (*p* = 0.734) across the three gestational trimesters (Figure 1). There was only a 2.6% increase in the proportion of pregnant women with adequate knowledge from the first to the third gestational trimester (Figure 1).

### 3.3. Factors Associated with a Lack of Adequate Knowledge on Mode of Delivery

In the univariate logistic regression model, pregnant women who were 18–24 years old (OR, 1.61, 95% confidence interval (CI), 1.07–2.44), underwent CS delivery in the previous pregnancy (OR, 1.81, 95% CI, 1.18–2.75), or preferred CS delivery for the current pregnancy (OR, 1.97, 95% CI, 1.14–3.38), were at a higher odds of having a lack of adequate knowledge on the mode of delivery. In the multivariate logistic regression mode, only pregnant women 18–24 years old (aOR, 3.07, 95% CI, 1.07–8.86) and those who had a CS delivery in a previous pregnancy (aOR, 1.90, 95% CI, 1.06–3.40), were more likely to be classified as having a lack of adequate knowledge on the mode of delivery (Table 3).

### 3.4. Preferred Mode of Delivery for Current Pregnancy

Of the 1250 pregnant women who replied to the question inquiring about the preferred mode of delivery for their current pregnancy, 9.8% preferred undergoing CS over vaginal delivery. In the univariate analysis, elder women (25–29 years or ≥30 years) were more likely to prefer undergoing CS delivery (OR, 7.91, 95% CI, 1.85–33.79, and OR, 14.82, 95% CI, 3.62–60.67, respectively). Being currently employed (OR, 1.84, 95% CI, 1.24–2.72), having at least one child (OR, 3.27, 95% CI, 1.68-6.35) or having been pregnant at least once before (OR, 2.28, 95% CI, 1.39–3.75), having their last birth delivered by CS (OR, 30.53, 95% CI, 18.02–51.73), and having a lack of adequate knowledge on the mode of delivery (OR, 1.97, 95% CI, 1.14–3.38), were all significantly associated with preference towards CS delivery. In the multivariate model, only currently employed women (aOR, 1.96, 95% CI, 1.13-3.40) and women who had their last birth by CS delivery (aOR, 31.10, 95% CI, 17.71–55.73) retained significant association (Table 4).

## 4. Discussion

The study has shown that the majority of pregnant women may not fully appreciate the potential health risks associated with different modes of delivery. The lack of adequate knowledge was more concentrated among young pregnant women and surprisingly among women who had their last birth by CS delivery. Adequacy of knowledge was not affected by the pregnancy trimester. The current study also showed that the CS mode of delivery was preferred by approximately 10% of pregnant women, particularly older pregnant women who were employed, multiparous, or those who had their last birth by CS. The WHO addressed the alarming rise of CS rates in both developed and developing countries [2], indicating that the practice of CS is not influenced by the economic conditions of the country and is rather a socio-cultural and institutional issue [21]. Pregnant women with a lack of adequate knowledge may accept the physicians’ recommendations without discussing possible alternative modes of delivery or understanding the risks associated with CS delivery [22,23]. Non-clinical educational interventions was the top recommendation by the WHO to halt the growing rise in CS rate [24]. Indeed, empowering pregnant women with the correct knowledge enhances their involvement in the decision-making process towards mode of delivery, and encourages a positive attitude towards vaginal delivery [25].

The inverse association between young age and lack of knowledge could be attributed to the fact that young women are less likely to experience adverse maternal outcomes that could increase their knowledge. The noteworthy finding is the positive association between a lack of adequate knowledge on mode of delivery and the lifetime experience of CS delivery. Women who had their last birth by CS delivery are theoretically and practically more likely to be informed on the reasoning, and more likely to be educated about the outcomes associated with CS delivery. This finding implies that primiparous women are not sufficiently exposed to the necessary information related to the mode of delivery. On the other hand, women with past CS delivery had a greater preference for CS, and this can be explained by the fact that a CS may help women plan their maternity leave, and allow them to have a more organized delivery plan. Another explanation may be related to the lack of past complications experienced in CS, thereby influencing a woman’s decisions and beliefs that all CS deliveries are safe or risk-free. Other women’s experiences, and cultural influences in the UAE may also play a role in mode of delivery decisions. Further exploration is needed to understand these findings. This potential explanation could be justified by the finding that a substantial proportion of pregnant women thought that CS is less painful and without as many complications. These findings match that of a study conducted in India which showed that pregnant women who preferred CS believed that CS delivery was less painful and less dangerous [26]. Further, Rice et al., reported that Thai women in Australia thought that CS delivery was a safe procedure and less painful [27]. Further investigation and exploration is needed, possibly using a qualitative or mixed-methods approach, to understand the factors that may lead women who experienced past vaginal deliveries and CS delivery to opt for CS delivery for their current pregnancy.

Pregnant women in their second and third trimester are more likely to have more ANC visits. An interesting finding in our study is the observed consistent low proportion of pregnant women with a lack of adequate knowledge on the mode of delivery across the three trimesters. The possible explanation for this observation may be that many of the participants were recruited in their third trimester (54.5%, Table 1) and therefore had initiated their ANC visits at a later stage as reported by Ali et al. [28]. Interestingly, a recent publication from our research group showed that 13.5% of women (N = 234/1737) in the Mutaba’ah Study had a history of two or more consecutive miscarriages, and that women with a history of recurrent miscarriage were twice as likely to undergo CS [29]. Future work would do well to explore the impact of infertility and recurrent miscarriages on choice of delivery mode if there is a preconception that CS deliveries are safer than vaginal deliveries amongst this study population.

Exploring women’s knowledge on mode of delivery in the UAE will be paramount in paving the way towards evidence-based policies and guidelines that might regulate the overall CS rate in the country. The study findings indicate that efforts to decrease the rates of CS need to incorporate stronger policies and guidelines supporting maternal education on the potential short and long-term health risks to the mother and child associated with CS, especially multiple CS. Furthermore, concerted actions to incorporate ANC appointments with a balanced discussion on the potential benefits and harms associated with different delivery modes are warranted.

The cross-sectional nature of the collected data limits identifying causality pathways between the factors associated with a lack of adequate knowledge and the mode of delivery. The arbitrary categorization of the knowledge into lack of and adequacy of knowledge might have introduced bias to the level of knowledge. However, categorization of the knowledge score into poor/moderate (score 0–6) or high (score 7–10) as previously reported [19] produced only 9.1% of the women with high knowledge score. This analysis might also be limited by the basic structure of the knowledge-related statements inquiring about the mode of delivery and by the self-reported data. Surveying a representative sample of women from the Emirati population from only one city in the UAE limits the generalizability of our findings to all pregnant women in the UAE.

Despite these potential limitations, the present study provides an important snapshot on the status of pregnant women’s knowledge about the risks and benefits of different modes of delivery. The high proportion of pregnant women with a lack of adequate knowledge might affect the patient-centered care and patient involvement in the decision-making process regarding the mode of delivery. The observed unchanged level of knowledge across the pregnancy trimesters sheds an important light on the importance of educating mothers and increasing their knowledge about the risks and benefits and indications of different delivery routes. Increased health literacy about decision-making with respect to modes of delivery is prudent in women having informed choices about their reproductive health. Empowering women by providing them with knowledge and skills which would allow them to make informed decisions is key in any health system. Providing women with more information on CS, the potential harms and needs of CS, as well as the clinical indications of CS delivery during additional prenatal or antenatal sessions may help to empower women with the necessary knowledge to make a better-informed decision regarding their birth plans. Such structured classes can be beyond, and external to, normal clinical appointments. Additionally, online reproduction-related communities have been shown to allow for narratives with regard to sharing childbirth information [30]. Moreover, pre-marital screening is compulsory for UAE national couples which presents an optimal opportunity to share recommendations well before conception periods [31].

## 5. Conclusions

This study illustrates the lack of adequate knowledge among pregnant women on the potential health risks associated with different modes of delivery, and that the level of knowledge was not affected by advanced gestational age. Approximately one out of ten pregnant women prefer to undergo CS delivery. Antenatal care appointments should include a more balanced discussion on the potential benefits and harms associated with different delivery modes to improve maternal knowledge about CS, and guide pregnant woman to make informed choices regarding their mode of delivery. There is a need to formulate innovative health policies towards women empowerment with appropriate knowledge related to the delivery modes.

## Figures and Tables

**Table 1:** Sociodemographic and maternal characteristics of pregnant women (N = 1303).

	N (Valid %)
**Age** (mean = 30.6 years ± 5.8 SD)	
18–24	209 (16.0)
25–29	350 (26.9)
≥30	744 (57.1)
**Educational attainment**	
Two-year diploma and above	639 (53.1)
Secondary schooling and below	565 (46.9)
Missing	99
**Husband’s educational attainment**	
Two-year diploma and above	628 (50.2)
Secondary schooling and below	623 (49.8)
Missing/Do not know	52
**Employed**	
No	830 (68.9)
Yes	374 (31.1)
Missing	99
**Gestational trimester** (mean gestational age = 6.4 months ± 2.2 SD)	
First	184 (14.1)
Second	409 (31.4)
Third	710 (54.5)
**Parity** (mean = 3.0 ± 1.8 SD)	
Nulliparous (1st pregnancy)	279 (23.4)
At least one child	913 (76.6)
Missing	111
**Gravidity** (mean = 3.6 ± 2.2 SD)	
Never been pregnant before	394 (30.2)
At least one pregnancy	909 (69.8)
**Mode of last delivery**	
Vaginal	772 (79.2)
CS	203 (20.8)
Missing/Nulliparous	328
**Previous eventful pregnancy**	
Never	199 (23.1)
1–2 events	494 (57.4)
≥3 events	168 (12.9)
Missing/nulliparous	442
**Preferred mode of upcoming delivery**	
CS	122 (9.4)
Vaginal	1128 (86.9)
Have not decided	48 (3.6)
Missing	5

SD: standard deviation, CS: cesarean section.

**Table 2:** Measured maternal knowledge using the ten knowledge-based statements inquiring about the mode of delivery.

Knowledge Item	Expected Answer	Reported Answer	
		Correct	Incorrect or Not Sure
		n (%)	n (%)
Cesarean delivery is less painful	Strongly agree/agree	311 (23.9)	992 (76.1)
Maternal complications of cesarean delivery are greater	Strongly agree/agree	813 (62.4)	490 (37.6)
Infection risk of cesarean delivery is higher than vaginal delivery	Strongly agree/agree	599 (46.0)	704 (54.0)
Emotional relationship between mother and baby after vaginal delivery is better	Strongly agree/agree	824 (63.2)	479 (36.8)
Infants born by cesarean section are smarter compared with those born by vaginal delivery	Strongly disagree/disagree	493 (37.8)	810 (62.2)
Infant bone fractures are impossible in cesarean section delivery	Strongly disagree/disagree	219 (16.8)	1084 (83.2)
It is reasonable to request cesarean section again for next delivery after the first cesarean section	Strongly agree/agree	432 (33.2)	871 (66.8)
Respiratory disorders in infants born by cesarean section are less likely than those born by vaginal delivery	Strongly disagree/disagree	245 (18.8)	1058 (81.2)
Hemorrhage after cesarean delivery is less than after vaginal delivery	Strongly disagree/disagree	210 (16.1)	1093 (83.9)
Cesarean section is reasonable when the baby is in breech presentation	Strongly agree/agree	771 (59.2)	532 (40.8)
Adequacy of knowledge (mean score = 3.7 ± 2.2 SD)			
Lack of adequate knowledge (score 0–5)	1022 (78.4)		
Adequate knowledge (score 6–10)	281 (21.6)		

**Table 3:** Univariate and multivariate sociodemographic and maternal characteristics associated with a lack of adequate knowledge (score = 0–5) on CS delivery.

	OR (95% CI)	aOR (95% CI)
**Age, years**		
≥30	1.00	1.00
25–29	0.97 (0.72–1.31)	0.90 (0.61–1.33)
18–24	1.61 (1.07–2.44) ^1^	3.07 (1.07–8.86) ^1^
**Educational attainment**		
Two-years diploma or above	1.00	1.00
Secondary schooling or below	1.31 (0.99–1.73)	1.34 (0.95–1.89)
**Husband’s educational attainment**		
Two-years diploma or above	1.00	1.00
Secondary schooling or below	1.06 (0.81–1.38)	1.08 (0.79–1.31)
**Current working status**		
Working	1.00	1.00
Not working	1.18 (0.88–1.58)	1.12 (0.85–1.59)
**Gestational trimester**		
Third	1.00	1.00
Second	0.93 (0.69–1.26)	0.71 (0.48–1.04)
First	0.86 (0.58–1.27)	0.75 (0.46–1.24)
**Parity**		
At least one child	1.00	1.00
Nulliparous	1.19 (0.85–1.68)	–
**Gravidity**		
Never been pregnant before	1.00	1.00
At least one pregnancy	0.99 (0.75–1.33)	–
**Previous eventful pregnancy**		
≥3 events	1.00	1.00
1–2 events	1.33 (0.88–2.01)	1.36 (0.88–2.12)
Never	1.28 (0.79–2.1	1.39 (0.88–2.37)
**Mode of last delivery**		
Vaginal	1.00	1.00
CS	1.81 (1.18–2.75) ^2^	1.90 (1.06–3.40) ^1^
CS, (after excluding nulliparous women)	1.89 (1.13–3.16) ^1^	2.15 (1.22–3.79) ^2^
**Preferred mode of delivery for current pregnancy**		
Vaginal	1.00	1.00
CS	1.97 (1.14–3.38) ^1^	1.41 (0.66–3.00)
Have not decided yet	2.08 (0.87–4.94)	1.02 (1.04–3.33)

CS: cesarean section; OR: odds ratio; adjusted odds ratio for age (as continuous for other variables), gestational trimester, women’s education, previous eventful pregnancies, and last birth delivery by CS. ^1^ *p* < 0.05, ^2^ *p* = 0.001.

**Table 4:** Distribution of pregnant women according to preferred mode of delivery and factors associated with opting for CS delivery.

	CS 122 (%)	Vaginal 1128 (%)	*p*-Value	OR (95% CI)	aOR (95% CI)
**Age**			<0.001		
18–24	95 (77.9)	625 (55.4)		1.00	1.08 (1.02–1.14) ^2^
25–29	25 (20.5)	308 (27.3)		7.91 (1.85–33.79) ^3^	
≥30	2 (1.6)	195 (17.3)		14.82 (3.62–60.67) ^4^	
**Educational attainment**			0.811		
Two-years diploma and above	59 (51.8)	551 (52.9)		1.00	–
Secondary schooling and below	55 (48.2)	490 (47.1)		1.05 (0.71–1.54)	
**Husband’s educational attainment**			0.265		
Two-years diploma and above	53 (44.9)	545 (50.3)		1.00	–
Secondary schooling and below	65 (55.1)	538 (49.7)		1.24 (0.85–1.82)	
**Employed**			0.004		
No	65 (57.0)	738 (70.9)		1.00	1.00
Yes	49 (43.0)	303 (29.1)		1.84 (1.24–2.72) ^3^	1.96 (1.13–3.40) ^2^
**Gestational trimester**			0.462		
First	16 (13.1)	159 (14.1)		1.00	–
Second	44 (36.1)	345 (30.6)		1.26 (0.70–2.14)	
Third	62 (50.8)	624 (55.3)		0.99 (0.56–1.76)	
**Parity** ^1^			<0.001		
Nulliparous	10 (8.8)	248 (24.1)		1.00	
At least one child	103 (91.2)	782 (75.9)		3.27 (1.68–6.35) ^4^	1.60 (0.30–8.37)
**Gravidity** ^1^			0.001		
Never been pregnant before	20 (16.4)	349 (30.9)		1.00	1.00
At least one pregnancy	102 (83.6)	779 (69.1)		2.28 (1.39–3.75) ^2^	1.38 (0.50–3.84)
**Mode of last delivery**			<0.001		
Vaginal	20 (18.7)	737 (87.5)		1.00	1.00
CS	87 (81.3)	105 (12.5)		30.53 (18.02–51.73) ^4^	31.10 (17.71–55.71) ^4^
**Knowledge level**			0.013		
Adequate knowledge	16 (13.1)	258 (22.9)		1.00	1.00
Lack of adequate knowledge	106 (86.9)	870 (77.1)		1.97 (1.14–3.38) ^2^	1.70 (0.81–3.57)

CS: cesarean section; OR: odds ratio; aOR: adjusted odds ratio for age (as continuous for other variables), employment, and parity, and last birth delivery by CS. ^1^ adjusted separately due to collinearity between parity and gravidity. ^2^ *p* < 0.05, ^3^ *p* = 0.001, ^4^ *p* < 0.001

## References

[1] Betrán A.P., Torloni M.R., Zhang J., Ye J., Mikolajczyk R.T., Deneux-Tharaux C., Oladapo O.T., Souza J.P., Tunçalp Ö., Vogel J.P. (2015). What is the optimal rate of caesarean section at population level? A systematic review of ecologic studies. Reprod. Health.

[2] WHO World Health Organization Statement on Caesarean Section Rates. http://apps.who.int/iris/bitstream/10665/161442/1/WHO_RHR_15.02_eng.pdf.

[3] Steer P.J., Modi N. (2009). Elective caesarean sections—Risks to the infant. Lancet.

[4] Victora C.G., Barros F.C. (2006). Beware: Unnecessary caesarean sections may be hazardous. Lancet.

[5] Mascarello K.C., Matijasevich A., Santos I., Silveira M.F. (2018). Early and late puerperal complications associated with the mode of delivery in a cohort in Brazil. Rev. Bras. Epidemiol..

[6] Sharma S., Dhakal I. (2018). Cesarean vs Vaginal Delivery: An Institutional Experience. JNMA J. Nepal. Med. Assoc..

[7] Fahmy W.M., Crispim C.A., Cliffe S. (2018). Association between maternal death and cesarean section in Latin America: A systematic literature review. Midwifery.

[8] Zaręba K., Banasiewicz J., Rozenek H., Wójtowicz S., Jakiel G. (2020). Peripartum Predictors of the Risk of Postpartum Depressive Disorder: Results of a Case-Control Study. Int. J. Environ. Res. Public Health.

[9] Shay L.A., Lafata J.E. (2015). Where Is the Evidence? A Systematic Review of Shared Decision Making and Patient Outcomes. Med. Decis. Mak..

[10] Stiggelbout A.M., Pieterse A.H., De Haes J.C.J.M. (2015). Shared decision making: Concepts, evidence, and practice. Patient Educ. Couns..

[11] Veroff D., Marr A., Wennberg D.E. (2013). Enhanced Support For Shared Decision Making Reduced Costs Of Care For Patients With Preference-Sensitive Conditions. Health Aff..

[12] Weckesser A., Farmer N., Dam R., Wilson A., Morton V.H., Morris R.K. (2019). Women’s perspectives on caesarean section recovery, infection and the PREPS trial: A qualitative pilot study. BMC Pregnancy Childbirth.

[13] Majlesi M., Montazeri A., Rakhshani F., Nouri-Khashe-Heiran E., Akbari N. (2020). ‘No to unnecessary caesarean sections’: Evaluation of a mass-media campaign on women’s knowledge, attitude and intention for mode of delivery. PLoS ONE.

[14] UN The Millennium Development Goals Report. https://www.un.org/millenniumgoals/2015_MDG_Report/pdf/MDG%202015%20rev%20.

[15] WHO Countdown to 2015: Maternal, Newborn, & Child Survival. http://www.countdown2015mnch.org/.

[16] Taha Z., Hassan A.A., Wikkeling-Scott L., Papandreou D. (2019). Prevalence and Associated Factors of Caesarean Section and its Impact on Early Initiation of Breastfeeding in Abu Dhabi, United Arab Emirates. Nutrition.

[17] Al-Rifai R.H., Ali N., Barigye E.T., Al Haddad A.H.I., Al Maskari F., Loney T., Ahmed L.A. (2020). Maternal and birth cohort studies in the Gulf Cooperation Council countries: A systematic review and meta-analysis. Syst. Rev..

[18] Al Haddad A., Ali N., Elbarazi I., Elabadlah H., Al-Maskari F., Narchi H., Brabon C., Ghazal-Aswad S., Alshalabi F.M., Zampelas A. (2019). Mutaba’ah—Mother and Child Health Study: Protocol for a prospective cohort study investigating the maternal and early life determinants of infant, child, adolescent and maternal health in the United Arab Emirates. BMJ Open.

[19] Ghotbi F., Sene A.A., Azargashb E., Shiva F., Mohtadi M., Zadehmodares S., Farzaneh F., Yasai F.-A.-M. (2014). Women’s knowledge and attitude towards mode of delivery and frequency of cesarean section on mother’s request in six public and private hospitals in Tehran, Iran, 2012. J. Obstet. Gynaecol. Res..

[20] STROBE Group (2007). STROBE Statement: Home. https://www.strobe-statement.org/index.php?id=strobe-home.

[21] Jadoon B., Mahaini R., Gholbzouri K. (2019). Determinants of over and underuse of caesarean births in the Eastern Mediterranean Region: An updated review. East. Mediterr. Health J..

[22] Bergeron V. (2007). The ethics of cesarean section on maternal request: A feminist critique of the american college of obstetricians and gynecologists’ position on patient-choice surgery. Bioethics.

[23] Minkoff H. (2006). The Ethics of Cesarean Section by Choice. Semin. Perinatol..

[24] WHO World Health Organization Recommendations: Non-Clinical Interventions to Reduce Unnecessary Caesarean Sections. https://www.who.int/reproductivehealth/publications/non-clinical-interventions-to-reduce-cs/en/.

[25] Eide K.T., Bærøe K. (2020). How to reach trustworthy decisions for caesarean sections on maternal request: A call for beneficial power. J. Med. Ethic.

[26] Saoji A., Nayse J., Kasturwar N., Nisha R. (2011). Women’s knowledge, perceptions, and potential demand towards caesarean section. Natl. J. Community Med..

[27] Rice P.L., Naksook C. (1998). Caesarean or vaginal birth: Perceptions and experience of Thai women in Australian hospitals. Aust. N. Z. J. Public Health.

[28] Ali N., Elbarazi I., Alabboud S., Al-Maskari F., Loney T., Ahmed L.A. (2020). Antenatal Care Initiation among Pregnant Women in the United Arab Emirates: The Mutaba’ah Study. Front. Public Health.

[29] Ali N., Elbarazi I., Ghazal-Aswad S., Al-Maskari F., Al-Rifai R.H., Oulhaj A., Loney T., Ahmed L.A. (2020). Impact of Recurrent Miscarriage on Maternal Outcomes in Subsequent Pregnancy: The Mutaba’ah Study. Int. J. Women’s Health.

[30] Fransisco K., Sanchez M. (2017). Vaginal Birth After Cesarean Section (VBAC): Informed Choice and a Source of Empowerment among Black Women in the United States. Global Perspectives on Women’s Sexual and Reproductive Health across the Lifecourse.

[31] Abu Dhabi Department of Health (AKA Health Authority), Health Authority-Abu Dhabi (2013). HAAD Standard for Premarital Screening and Counseling Program. Doh.gov.ae.

